# Acetylation and Evaluation of Taro Boloso-I Starch as Directly Compressible Excipient in Tablet Formulation

**DOI:** 10.1155/2020/2708063

**Published:** 2020-03-12

**Authors:** Afewerk Getachew, Zewdu Yilma, Solomon Abrha

**Affiliations:** ^1^Department of Pharmaceutics, School of Pharmacy, College of Health Sciences, Mekelle University, P.O. Box 1871, Mekelle, Ethiopia; ^2^Department of Pharmacy, College of Medicine and Health Sciences, Bahir Dar University, P.O. Box 79, Bahir Dar, Ethiopia

## Abstract

Taro Boloso-I (TB1), a newly improved *Colocasia esculenta* variety, is a potential source of starch with high yield. However, to improve some limitations of the native starches (NS), such as flowability and compactibility, different physical and chemical starch modifications have been employed. Acetylation is one of the chemical modifications which improves the flow and compaction of the NS, which are prerequisite during direct compression (DC) of tablets. Hence, in this study, TB1 starch was acetylated using acetic anhydride and evaluated as an ideal excipient for direct compression. Starch acetates (SA) with a degree of substitution (DS) of 0.072 (SA_1_) and 0.695 (SA_2_) were produced and evaluated. FTIR spectra of the SAs were used to verify the acetylation of the NS. Powder flow evaluation parameters showed significant improvement in the flow properties of the NS following acetylation. In addition, the swelling power, solubility, and compactibility were also improved. Tensile strength (TS) of the tablets comprising SAs only, SA_1_ (41.40) and SA_2_ (63.43 Kg/cm^2^), was significantly higher than tablets made of the NS (31.96) and Starch 1500® (15.12 Kg/cm^2^). The SAs also showed lower sensitivity towards lubrication than the NS and Starch 1500® as lower lubricant sensitivity ratios were recorded. In addition, tablets comprising the SAs satisfactorily accommodated at least up to 50 % w/w paracetamol—compared to 30 % w/w by Starch 1500®—upon DC processing. The paracetamol tablets comprising SAs also complied with the United States Pharmacopeia specifications for disintegration and dissolution studies. Therefore, taking all the facts into consideration, the SAs could be potential DC excipients in tablet formulations.

## 1. Introduction

The rapidly evolving and competitive pharmaceutical market is promoting the production of innovative, high-quality, and low-cost products. Such requirements necessitate the development of new excipients with multifunctional properties [1]. Such excipients can provide pharmaceutical manufacturers with cost savings in drug development [2] and hence reduce the final cost of production. Many of these multifunctional excipients are produced through the modification and altering certain properties of the existing original types of material [1]. There is a continuous need for new starch excipients with desirable properties for pharmaceutical applications. Consequently, more attention has been focused on the development of starch from different botanical sources for this purpose [3]. In this regard, many tropical countries have different species which might be a potential source of starch [4]. For these sources to become competitive in the market, a significant amount of work remains to be done on the functional characteristics of the native as well as the modified ones.

**Figure 1:**
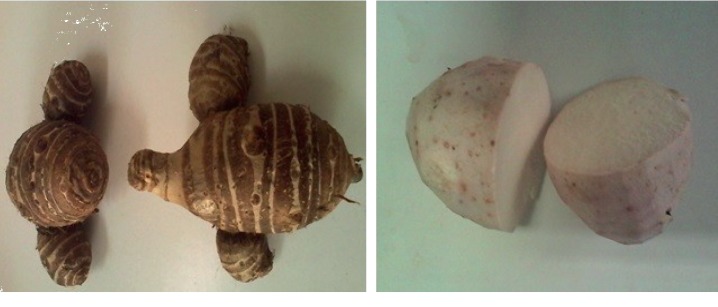
Image of Taro Boloso-I tuber (left) and the peeled tuber (right) (Photograph by Afewerk Getachew).

Among the nonconventional new sources of starch are tubers such as Taro [5] which is underutilized potential root crop in Ethiopia [6]. Taro is an erect herbaceous perennial root crop widely cultivated in the tropical and subtropical world belonging to genus *Colocasia* in the plant family called Araceae. Taro Boloso-I (TB1) is an improved new variety of *Colocasia esculenta,* officially released from Areka Agricultural Research Institute. It was developed from different accessions of Taro in terms of yield and relative resistance towards major diseases and pests [7]. The tubers of TB1 are depicted in Figure 1. Native TB1 starch (NTB1S), as indicated in a study [8], had high starch yield (around 83.5% on a dry weight basis), amylose content of around 20.7%, granule size less than 6.63 *μ*m, and good compaction property. However, its poor flow restricts its use as direct compression (DC) excipient in tablet formulations.

Most studies on chemical modification of starches have been limited to widely available starches such as maize, potato, and wheat. However, chemical modification of starches from other botanical sources may yield starches with functional properties desirable in the pharmaceutical industries. Furthermore, the derived starches may even have better properties as tablet excipients, especially in DC tablet manufacturing [9]. A DC excipient should have good binding functionality, good powder flowability, good compressibility, and high-dilution potential (DP) [10]. Chemically modified starches were found to achieve the desired DC properties for tablet manufacturing. Of those chemical modifications, starch acetylation was carried out in a simple manner that imparted improvement in the physicochemical and functional properties of native starches (NSs), even at a low degree of substitution (DS) [11]. In addition, starch acetates (SAs) with DS 0.01–0.2 do not require regulatory approval since they are approved by the FDA for food use [12, 13]. SAs with low DS have been applied in areas such as film former, binder, thickener, stabilizer [12, 13], disintegrant, and filler [14]. The present study aims to evaluate acetylated TB1S as a DC excipient for immediate release of solid-dosage forms.

## 2. Materials and Methods

### 2.1. Materials, Chemicals, and Reagents

Taro Boloso-I tubers were obtained from Areka Agricultural Research Institute located at Areka city, Ethiopia. Acetic anhydride (May and Baker Ltd., Dagenham, England), ethanol 96 % (Fine Chemical, Addis Ababa, Ethiopia), hydrochloric acid (Carlo Erba Reagents, Val-de-Reuil, France), magnesium stearate (UNI-CHEM, Goa, India), potassium hydroxide (BDH Chemicals Ltd., Pool, England), sodium chloride and sodium hydroxide (Abron Chemicals, Ambala, India), and Starch 1500® (Colorcon, Bougival, France) were used as received. The active ingredient, Paracetamol (Anhuibbca Linkang, Pharmaceutical Co., Ltd., Hangzhou, China), was kindly donated by Addis Pharmaceutical Factory (APF), Ethiopia. All the reagents and solvents used were of analytical grade.

## 3. Methods

### 3.1. Starch Isolation

NTB1S was extracted by a combination of the methods described by [8, 15]. First, fresh TB1 tubers were cleaned, peeled, and shredded into small thin slices and crushed with 1% NaCl solution using a blender. The resulting mass was then placed over a muslin cloth and repeatedly washed with a solution containing 1% NaCl and 0.03 N NaOH. The resulting sediment from the filtrate was further washed with demineralized water (DW) until the supernatant becomes clear and pH is neutral. The starch was then dried at 40°C in an oven (Memmert SM-200, Germany).

### 3.2. Acetylation of Starch

Acetylation was conducted as per the method described by [14, 16]. The starch sample (oven-dried at 50°C for 24h) was first mixed with acetic anhydride for five minutes (at 100 rpm stirring) in a 2 L oil bath jacketed glass reactor at room temperature. Aqueous NaOH solution (50% w/w) was then added dropwise. Then, the acetylation was carried out at reaction conditions I (at room temperature for 12 min) and II (at 90°C for 60 min). These conditions were used to obtain SAs with low and medium DS using starch:acetic anhydride:NaOH solution (50% w/w) ratio of 1 : 4:0.2 for both reaction conditions. At the end of the reaction time, the process was terminated by adding excess cold DW to the reactor with vigorous mixing. The precipitate formed was filtered using a suction filter and washed with DW several times. The SAs were then dried in an oven (Kottermann® 2711, Germany) at a temperature of around 40°C for 24 h. Then, it was milled, sieved, and stored in a glass container for further analysis.

### 3.3. Determination of Degree of Substitution

The DS was determined by saponification titration method [17, 18]. SA (1 g) was poured into a 250 mL flask, 50 mL ethanol (75 %) was added, and the slurry was kept in a water bath (Logan Instruments Corp., England) at 50°C for 30 min. Then, 40 mL of 0.5 N KOH was added after the slurry was cooled. The flask was then tightly covered with aluminum foil and left for 72 h for complete saponification. Then, excess alkali was back-titrated with 0.5 N HCl using phenolphthalein as an indicator. The solution was allowed to stand for an extra 2 h and titrated in case of additional alkali leaching out from the sample. A blank test was also carried out using the original unmodified NS. Then, the acetyl content (%*A*) and DS were calculated according to the following equations:

(1) $$\begin{array}{c} \%A=\frac{\left[\left(V_{b}-V_{s}\right)\times N\times 0.043\right]}{M}\times 100, \end{array}$$

(2) $$\begin{array}{c} DS=\frac{162\times \%A}{\left[4300-\left(42\times \%A\right)\right]}, \end{array}$$

where *V*_*b*_ and *V*_s_ are the volume (mL) of HCl consumed by the NS and the sample, respectively, *M* is the mass (*g*) of the sample used, and *N* is the normality of the HCl used for titration.

### 3.4. Identification Studies

Acetylation, the introduction of an acetyl group into the starch molecule, was confirmed using FTIR (Shimadzu-Prestige-21, Japan). First, a finely ground sample was mixed with liquid paraffin (a mulling agent) using mortar and pestle. The mixture was then sandwiched between KBr plates, placed in the IR spectrophotometer and the spectra were obtained. Each IR spectrum was collected with 20 scans and a spectral resolution of 2 cm^−1^. Scanning was performed between wavenumbers of 4000 and 400 cm^−1^. The background spectrum was also collected before running the sample.

### 3.5. Determination of Solubility and Swelling Power

The method used by Bello-Perez and his colleagues was employed for the determination of the percent solubility (S (%)) and swelling power (SP) of the samples [19]. 0.5 g of samples was dispersed in 10 mL of DW in centrifuge tubes (preweighed). Then, the tubes were transferred into a thermostatically controlled water bath for 30 min at 20, 37, 50, 65, 75, and 85°C, with shaking every 5 min, and then left to cool down. Then, the suspensions were placed in a centrifuge machine (Table Top Centrifuge, PLC 03, Taiwan), operated at 3000 rpm for 15 min and the supernatants were decanted onto dried and preweighed Petri dishes and dried in an oven for 2 h at 130°C. The S (%) and SP were calculated using the following equations:

(3) $$\begin{array}{c} S\left(\%\right)=\frac{W^{S}}{\text{WS}}\times 100, \end{array}$$

(4) $$\begin{array}{c} \text{SP}=\frac{W_{P}}{\text{WS}\times \left(100-S\right)}\times 100, \end{array}$$

where *W*^*S*^ is the weight (*g*) of soluble material in the supernatant, *W*_*P*_ is the weight (*g*) of the precipitate, and WS is the weight (*g*) of the starch sample.

### 3.6. Moisture Sorption Pattern

Moisture sorption patterns of NTB1S and TB1SA samples were determined by using the method described by [9]. Starch samples were dried in an oven for 4h at 120°C and spread evenly on dry preweighed Petri dishes. These Petri dishes were then transferred to particular RH chambers (i.e., 100, 75.6, 60, 40, and 20 RH) in different desiccators. The samples were left for 4 weeks to equilibrate at room temperature and then their weights were measured and recorded. The moisture uptake of each sample was calculated on the basis of their weight difference before and after equilibrium in a given RH. The water sorption capacity was then reported as percent moisture uptake.

### 3.7. Flow Characterization

For the determination of density related properties, a 30 g weight of the sample was weighed and poured into a 250 mL graduated measuring cylinder slowly at 45° and the bulk volume was determined. Then, it was placed on a tap densitometer (ERWEKA, SVM 223, Germany) and the taped volume was recorded after 500 taps and used to calculate the bulk and tapped density. Then, Hausner's ratio (HR) and Carr's index (CI) were calculated using the following equations:

(5) $$\begin{array}{c} \text{HR}=\frac{\rho_{t}}{\rho_{b}}, \end{array}$$

(6) $$\begin{array}{c} \text{CI }=\frac{\left(\rho_{t}-\rho_{b}\right)}{\rho_{t}}\times 100, \end{array}$$

where *ρ*_*t*_ and *ρ*_*b*_ are the tapped density and bulk density, respectively.

The flow rate and angle of repose of each sample were characterized employing a powder flow tester (PHARMA TEST PTG-S4, Germany). 30 g of each sample was filled into the apparatus, which contains a stainless steel conical funnel. Then, the machine was operated where the sample powders flow down 15 mm from outlet nozzle. Finally, the flow tester measured the flow rate and angle of repose of the samples.

### 3.8. Tablet Formulations

For the evaluation of compressibility, lubricant sensitivity (LS), and dilution potential (DP), 300 mg tablet formulations were prepared from SAs, NTB1S, and S1500®. All these tablet formulations were prepared using a tablet compression machine (MINI Press II, Germany) fitted with a flat-faced punch and die of 10 mm in diameter. The compression force was held constant at all times. The remaining specific details for each formulation and their composition are discussed in their respective sections below.

### 3.9. Compactibility and Lubricant Sensitivity

In the compactibility study of the above-listed powders, blank tablets of each powder were prepared without lubrication. Then, the tablet properties were evaluated after 24 h. To compare the effect of lubricant on the mechanical properties, blank tablets were prepared after each powder was lubricated with 2 % magnesium stearate (MgS) for 5 min. Then, the lubricant sensitivity ratio (LSR) was calculated using the following equation [20–22]:

(7) $$\begin{array}{c} \text{LSR}=\frac{\left({\text{TS}}_{0}-{\text{TS}}_{2}\right)}{{\text{TS}}_{0}}, \end{array}$$

where TS_0_ and TS_2_ are the tensile strength of tablet compacts prepared at 0 and 2% lubrication with MgS, respectively.

### 3.10. Dilution Potential

For the comparison of the DP of the concerned excipients, tablets containing paracetamol (PCM) were prepared at 20, 30, 40, and 50% content. PCM was first mixed with each powder for 10 min and then lubricated with 0.5 % MgS for 5 min. Four formulations were prepared for each excipient used in this study whose compositions are shown in Table 1.

### 3.11. Tablet Characterization

#### 3.11.1. Hardness and Friability

Crushing strength (Cs) of the prepared tablets was determined by compressing 10 randomly selected tablets diametrically on a tablet hardness tester (Pharma Test, PTB 311E, Hamburg, Germany). The results were recorded and reported as a mean value with their respective standard deviation. The radial tensile strength (TS) of the compacts was calculated from the Cs, diameter, and thickness of each tablet [23]. Friability was evaluated using Roche friabilator (ERWEK, Germany) operated at 25 rpm for 4 minutes using ten tablet samples.

#### 3.11.2. Disintegration Test

The disintegration times (DT) of selected tablets were determined in a disintegration apparatus (Pharma Test, PTZ-S, Hamburg, Germany) according to the disintegration test for uncoated tablets of the United States Pharmacopeia [24]. The disintegration medium was distilled water (900 ml) maintained at 37 ± 2.0°C.

#### 3.11.3. In Vitro Dissolution Studies

The in vitro dissolution studies were performed according to the USP (USP 30/NF 25, 2007) in a dissolution testing equipment (ERWEKA, Germany) using USP dissolution apparatus II (Paddle Method) adjusted to rotate at 50 rpm. 900 mL phosphate buffer solution (pH 5.8) was used as a dissolution medium maintained at 37 ± 0.5 °C. Then, a 5 mL sample of the dissolution medium was withdrawn at predetermined intervals (5, 10, 15, 20, 30, 45, and 60 min), filtered, appropriately diluted, and analyzed by UV/Visible spectrophotometer at *λ*_max_ of 243 nm.

#### 3.11.4. Statistical Analysis

Statistical analysis was performed using Analysis of Variance (ANOVA) with statistical software origin Pro 8 (Origin Lab ™ Corporation, USA). 95% confidence interval, *p* values less than 0.05 were considered statistically significant. All the results were reported as the mean and standard deviation.

## 4. Results and Discussion

### 4.1. Acetyl Content and Degree of Substitution

Two SA powders with DS of 0.072 (SA_1_) and 0.695 (SA_2_) were obtained after modification using reaction conditions I and II, respectively. The acetyl content and DS obtained are given in Table 2. Rincon-Aguirre et al. [5] acetylated Taro starch at 25°C for 30 min using the method of [25] and reported a DS of about 1.963. The difference with the present work might be attributed to the amylose content, which was reported to be about 15%. If that was the case, however, [26] using the method of [25] obtained a DS of 0.055 upon acetylation of Taro starch of a lower amylose content (around 2%). Such value was much lower than that reported by [5]. Nigussu et al. [14] acetylated enset starch at 90°C for 1 h using a method similar to the present study and obtained a DS of 0.672. The foregoing is comparable to the value obtained in this work for starch acetylation. However, a comparison of different studies would not be conclusive unless these procedures are made under similar conditions.

### 4.2. FTIR Identification Studies

The FTIR spectra of the NTB1S sample showed the same characteristic spectrum of a natural starch, i.e., a broadband at about 3350–3500 (–OH group stretching); strong peaks at about 2955, 2923, and 2854 cm^−1^ (related to C–H stretching); band at about 2727 and 2686 (–CH_2_ stretching); band at about 1631 (primary OH groups (CH_2_OH) deformation vibrations in water); the peaks at 1155 and 1080 (C–O–H bending); bands at 979, 933, and 862 (glycosidic linkage (C–O–C) skeletal mode vibration); and the bands between 765 and 520 cm^−1^ (pyranose ring skeletal mode vibration) [13, 22, 27–29]. Compared to NTB1S, a new absorption peak in the spectra of SA_1_ and SA_2_ was observed at about 1750 cm^−1^ whose intensity increased with the DS. This absorption peak is responsible for the stretching of an ester carbonyl (C=O) group [12, 30] and linearly related to the DS [31]. This indicated that NTB1S was successfully acetylated.

### 4.3. Powder Characterizations

#### 4.3.1. Density Related and Flow Properties

The powder properties of NTB1S, SA_1_, SA_2_, and S1500® are summarized in Table 3. From the results of the bulk density observed, the rank in ascending order was SA_2_ < SA_1_ < NTB1S < S1500®. Hence, a decrease in the bulk density was observed as the DS increased. This might be due to the introduction of (bulky) acetyl group which reduces the bond strength between starch molecules owing to steric hindrance. This leads to structural reorganization causing the opening up of the starch structure [11, 32, 33]. In general, the HR and CI show improvement upon modification by acetylation. However, this improvement was not significant for SA_1_ (*P* > 0.05) which may be attributed to the low DS. On the other hand, significant improvement was achieved for SA_2_ compared to the NS (*P* > 0.05). According to the HR and BD, the flow of S1500® was the highest compared to NTB1S, SA_1_, and SA_2_, which might be due to its higher bulk density. The flow rate of SA_1_ and SA_2_ indicated an improvement compared to NTB1S which was unable to flow through an orifice. This result was also supported by the values of AR indicating better flow. According to the flow rate and AR, the flow was ranked in the order of SA_1_ < S1500® < SA_2_. Such discrepancies in CI, HR, AR, and flow rate were reported by [36]. The flow- and density-related properties indicated that the flow of the NS was indeed improved by acetylation.

### 4.4. Moisture Sorption Pattern

**Figure 2:**
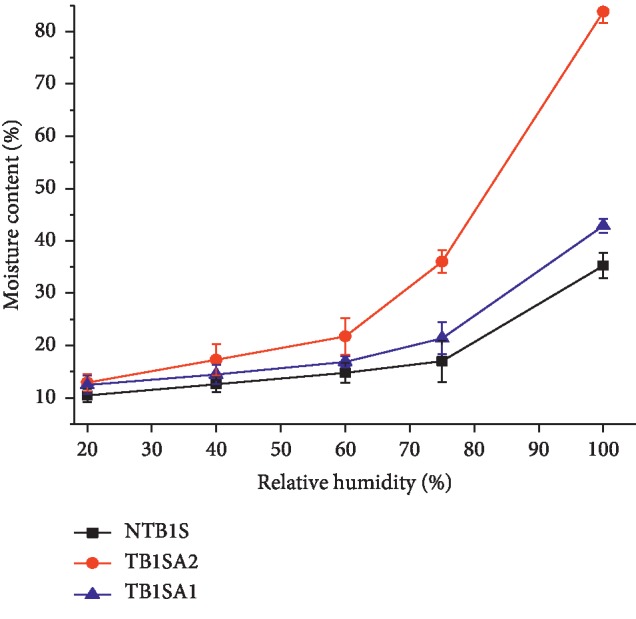
Moisture sorption patterns of NTB1S, SA_1_, and SA_2_ at room temperature.

The moisture sorption patterns of native and the modified starch samples are provided in Figure 2. The sorption property in descending order was SA_2_ > SA_1_ > NTB1S. Although the rank was in that manner, the difference between NTB1S and SA_1_ was not found to be significant except at the highest RH value (100). On the other hand, SA_2_ started to gain a significant amount of moisture and showed a significant difference from the other counterparts beyond the RH value of 60. The increased moisture sorption of SAs might be due to the introduction of acetyl groups into starch which could have facilitated the access of water to amorphous areas and increased water uptake [35]. Powder X-ray diffraction studies indicated that acetylation rendered NS into a more amorphous form and at higher DS; it could turn it into a completely amorphous material [36].

### 4.5. Swelling Power and Solubility

**Figure 3:**
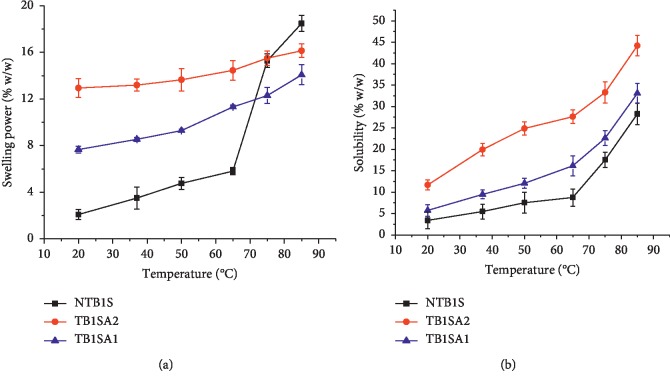
Swelling power (a) and solubility profiles (b) of NTB1S, SA_1_, and SA_2_.

The SP and S profiles of NTB1S, SA_1_, and SA_2_ are depicted in Figures 3(a) and 3(b), respectively. The SP was in increasing order of NTB1S < SA_1_ < SA_2_. Compared to the NTB1S, significant improvement in SP was observed for the SAs up to 65°C (*P* < 0.05). However, beyond this temperature, the SP significantly increased (*P* < 0.05) and became higher than SA_1_ and somewhat comparable with SA_2_. On the other hand, the SP of SA_2_ was superior to SA_1_ at all temperatures studied (*P* < 0.05). This might be attributed to the DS which introduces a higher level of granule disorganization at higher levels. The disruption of the granular and/or crystalline structure is responsible for the granule swelling and solubility [37]. The S profile in an increasing order was NTB1S < SA_1_ < SA_2_ with the latter having significantly higher S at all temperatures (*P* < 0.05) compared to all the previous ones. Similarly, the S of SA_1_ was also significantly higher than the NS at all temperatures except at 20°C and 85°C (*P* < 0.05). The NS showed lower solubility at a lower temperature since NS granules are insoluble in cold water. However, when the temperature was increased, S started to improve which might be due to the swelling of amorphous regions and the diffusion of mobile amylose molecules [38].

### 4.6. Compactibility Study

**Figure 4:**
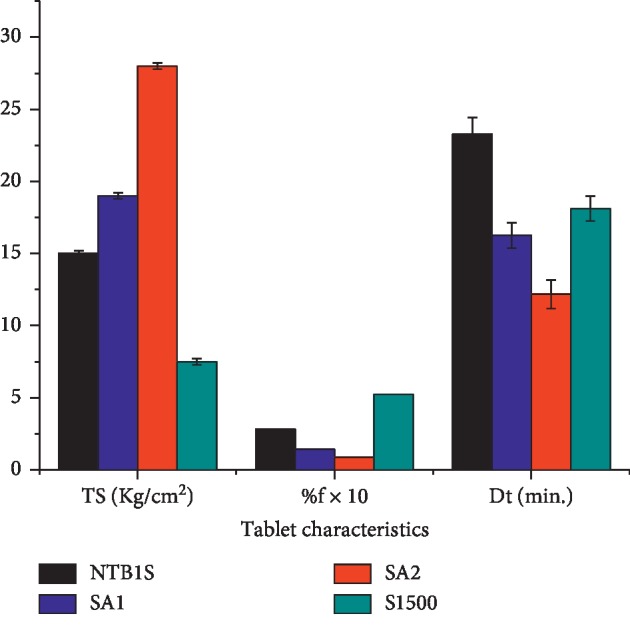
Tensile strength, friability, and disintegration time of blank tablets prepared from NTB1S, SA_1_, SA_2_, and S1500®.

Some tablet characteristics of the blank tablets prepared for the compactibility evaluation are presented in Figure 4. As can be clearly seen from this figure, SA_1_ and SA_2_ showed improved compaction compared to the NS and S1500® which was reflected in their higher TS. Generally, the ranking order of the TS of blank tablet was SA_2_ > SA_1_ > NTB1S > *S*1500® (*P* < 0.05). The higher compaction of SA_1_ and SA_2_ could be attributed to the modification as the TS increased significantly in line with the DS (*P* < 0.05). This might be due to the acetate moiety which is found to be a very effective bond former. It increases the formation of strong molecular bonds in combination with the existing hydroxyl groups. New molecular interactions, like van der Waals's forces, could also be involved. The strength of SA tablets might also be due to the enhanced plastic flow and slight fragmentation of the particles under compression which increases the bonding surface area. This all might lead to the formation of a very firm and intact tablet structure [39].

As depicted in Figure 4, the friability results of the blank tablets were in the ranking order of S1500® > NTB1S > SA_1_ > SA_2_ which was in line with their mechanical strength. All the blank tablets registered relatively lower friability which could be seen in their higher TS. However, SAs showed enhanced resistance for tablet weight loss as compared to the NS and S1500®. This could be attributed to the acetylation modification as the friability decreased with increasing DS.

The results for the disintegration test of the blank tablets (Figure 4) ranked in descending order were NTB1S < *S*1500® < SA_1_ < SA_2_. The DT of NTB1S was the longest which might be due to the appearance of a highly viscous structure upon swelling which hinders water penetration into the compact mass [40] and somewhat to its higher TS compared to S1500®. A similar argument could be put forward for the longer DT of S1500®—the formation of a gel-like layer [20]. In lieu of this, it might not be conclusive to compare the DT times of these blank tablets. However, a significant (*P* < 0.05) decline in the DT of SA_2_ as compared to SA_1_ could be noticed regardless of the highest TS registered by blank tablets of SA_2_. This might be the contribution of the improvement in the SP associated with the acetylation (see Figure 3 above). Generally, the longer DT registered by all the tablets may also be attributed to the MgS film formed on the tablet layer due to the external lubrication of the die and the punch.

### 4.7. Lubricant Sensitivity

**Figure 5:**
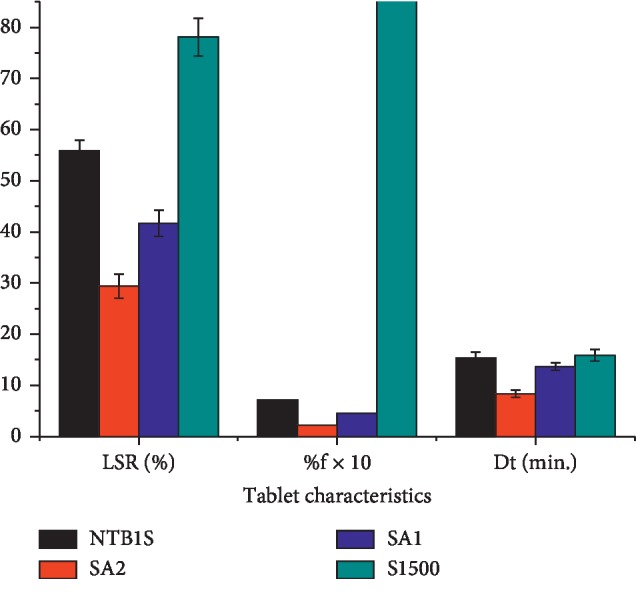
LSR, friability, and disintegration time of blank tablets prepared from NTB1S, SA_1_, SA_2_, and S1500® using 2% MgS.

One approach for the evaluation of materials' sensitivity to the addition of lubricants is the use of LSR [41]. The more this value approaches 1, the more the powder is sensitive to an added lubricant from the viewpoint of decreased mechanical strength [20]. As can be seen in Figure 5, the highest value of LSR was registered by S1500® which lost about 78.1 % of its initial TS while the lowest value was that of SA_2_ which had lost only about 29.3 % of its initial TS. Generally, the LSR significantly (*P* < 0.05) increased in the order of SA_2_ (0.293), SA_1_ (0.42), NTB1S (0.56), and S1500 (0.78). Based on these results, it could be said that the acetylation of the NS had rendered it less sensitive to MgS.

The friability and DT results of the blank tablets produced using 2% MgS are presented in Figure 5. Accordingly, the smallest friability was registered by SA_2_ (0.22) followed by SA_1_ (0.45) and NTB1S (0.72%). Hence, SA_2_, SA_1_, and NTB1S could at list accommodate up to 2% MgS as reflected in their resistance to friability. On the other hand, tablets of S1500® were with the highest friability value where most of the tablets were broken during the operation which is represented by a long and open bar in Figure 5. Regarding the DT,_,_ tablets of S1500® were normally expected to disintegrate faster considering their lowest TS. However, these tablets registered the highest DT which might be attributed to the formation of hydrophobic MgS film on the tablet surface limiting the access of water into the tablet [42, 43]. Regardless of their highest TS, tablets of SA_2_ registered the lowest DT followed by SA_1_ which might be attributed to their highest SP and lowest LS compared to the NTB1S.

### 4.8. Dilution Potential

#### 4.8.1. The Hardness and Friability of Paracetamol Tablets

**Figure 6:**
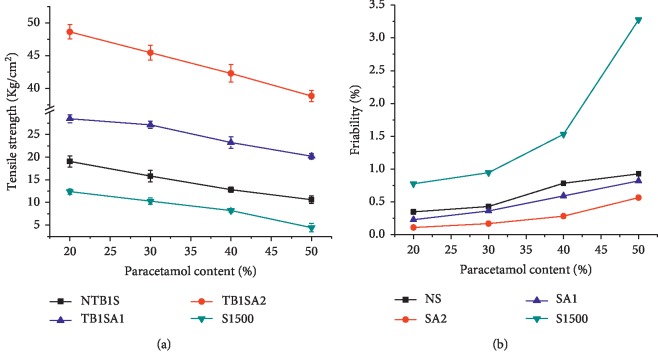
The tensile strength (a) and friability profile (b) of tablets formulated from NTB1S, SA_1_, SA_2_, and S1500® at different paracetamol loading.

Roughly *C*_S_ of about 50–80 N is acceptable for conventional tablets [44]. The tablets prepared from NTB1S, SA_1_, and SA_2_ were hard enough at all PCM contents having *C*_S_ of 54.2 ± 4.290, 96.4 ± 2.914, and 182.3 ± 3.908 N at 50% PCM content, respectively. With respect to this, tablets of S1500® were hard enough only up to 30% PCM loading. The relationship between PCM content and the TS of the tablets is depicted in Figure 6(a). This figure indicates that the TS of all tablet formulations generally declined with increasing PCM content within the tablets. This could be explained by the fact that increased PCM content enhanced the destructive components of the compact formation [45]. The highest TS was registered by the tablets of SA_2_ which was more than twofold of the NS. Similarly, SA_1_ tablets attained higher TS than the latter one. However, the TS of SA_1_ tablets was significantly inferior compared to SA_2._ The highest TS observed in the SAs could be attributed to the acetylation modification which could be strengthened by the fact that the increase in TS was in line with the DS. Generally, the TS of PCM tablets in an increasing order was S1500® < NTB1S < SA_1_ < SA_2_ (*P* < 0.05) at all levels of PCM content.

According to Figure 6(b), the friability of the tablet formulations was ranked in increasing order as SA_2_ < SA_1_ < NTB1S < *S*1500®. Generally, tablets prepared from NTB1S, SA_1_, and SA_2_ fulfilled the acceptance criteria at all PCM contents, while tablets of S1500® fulfilled this specification only up to 30 % PCM content. This could be due to its low tablet mechanical strength. There is a direct relationship between tablet mechanical strength and friability in such a way that the latter declines with the former increasing [46]. Hence, all the tablets formulations showed an increase in percent friability in line with the decrease in their TS associated with the increased PCM content.

#### 4.8.2. Disintegration Time

**Figure 7:**
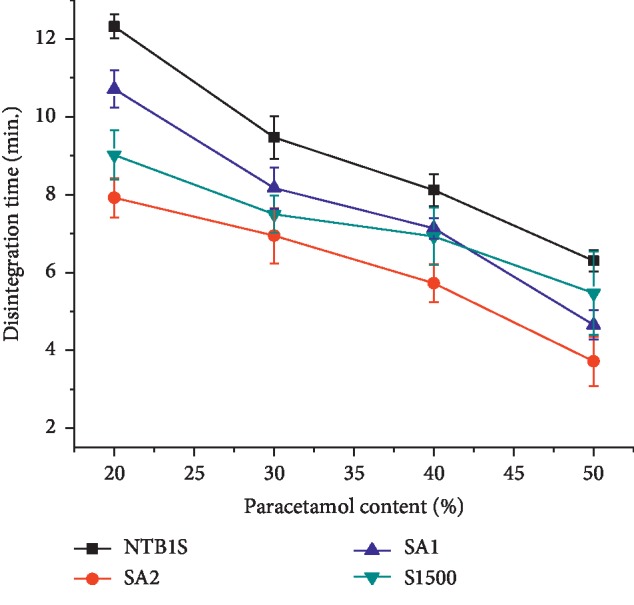
The disintegration time of tablets prepared from NTB1S, SA_1_, SA_2_, and S1500® at a different level of paracetamol.

It can be seen from Figure 7 that the DT of the PCM tablets showed a declining trend with an increasing PCM content (*P* < 0.05) which could be attributed to the poor compaction nature of PCM. This results in the weakness of the tablet mechanical strength while enhancing water penetration and the DT. Despite their higher TS compared to the tablets of NTB1S, tablets of SA_2_ and SA_1_ showed faster DT at all PCM contents. This could be attributed to their improved SP [47]. Generally, the DT was in the order of SA_2_ < S1500® < SA_1_ < NTB1S. The lower DT registered by tablets of S1500® could be as a consequence of their low mechanical strength. In spite of these differences, all PCM tablet formulations fulfilled the pharmacopoeial specification for the disintegration of conventional tablets (<15 min) (USP 30/NF 25, 2007).

#### 4.8.3. Dissolution Study of Paracetamol Tablets

**Figure 8:**
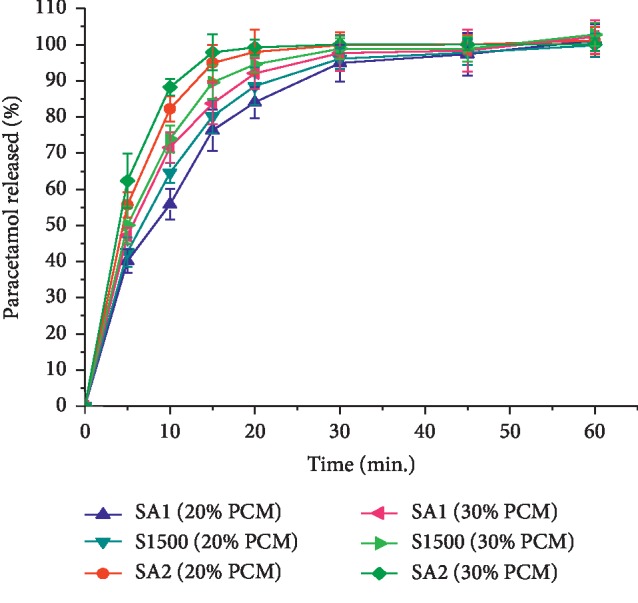
The dissolution profile of tablets prepared from SA_1_ SA_2_, and S1500® at 20% and 30% paracetamol content.

The dissolution profile of PCM tablets prepared using SA_1_, SA_2_, and S1500® as DC excipient at 20 and 30% loading was as depicted in Figure 8. The amount of PCM released within 30 min from tablets containing lower concentration of PCM was in the order of SA_2_ (99.9) > S1500® (96.1) > SA_1_ (94.9) (*P* < 0.05). This was in concordance with the DT registered by the respective tablets. Generally, incorporation of a relatively higher amount of PCM into the tablets increased its release at all times. This might be as a consequence of the decline in TS and DT of the tablets. The presence of drug molecules probably disturbs chain entanglement and weakens the network structure and hence the subsequent increase in drug diffusion [48]. However, drug release from tablets containing 30 % PCM at 30 min, with the rank order of SA_2_ > S1500® > SA_1_, was not found to be significant (*P* < 0.05). Regardless of the excipients used, all the tablets in this study fulfilled the USP specification for tablets; i.e., >80% tablet content should be released within 30 min (USP 30/NF 25, 2007).

## 5. Conclusions

In this study, SAs with DS of 0.072 and 0.695 were obtained using different acetylation conditions. The characterizations of these SAs indicated that the acetylation had resulted in improved physicochemical properties such as the SP, S, and flow properties. The compaction property of the SAs, in terms of the mechanical strength and resistance to friability, were found to be superior compared to both the NS and S1500®. Similarly, acetylation of the NTB1S improved its DP in line with the DS. That is, tablets made of the SAs were observed to possess the highest mechanical strength and lowest friability compared to the NS and S1500®. Therefore, taking all the findings into consideration, the SAs could be potential DC excipients, especially, SA_1_, the one with the lowest DS.

## Figures and Tables

**Table 1:** The composition of tablets formulated for the study of DP.

Formulation	PCM (%)	X (%)	MgS (%)
I	20	79.5	0.5
II	30	69.5	0.5
III	40	59.5	0.5
VI	50	49.5	0.5

X stands for the amount of the respective excipients used (NTB1S, SA_1_, SA_2_, and S1500®).

**Table 2:** The acetyl contents and degree of substitution obtained using reaction conditions I and II.

Reaction time (min.)	Acetyl content (%*A*)	Degree of substitution (DS)
I	1.86 ± 0.124	0.072 ± 0.005
II	15.6 ± 0.448	0.695 ± 0.024

Values are represented as SD.

**Table 3:** Some powder properties of native, modified starch and Starch 1500®.

Powders	NTB1S	SA_1_	SA_2_	S1500®
BD (g/mL)	0.453 ± 0.002	0.413 ± 0.004	0.309 ± 0.003	0.621 ± 0.010
TD (g/mL)	0.590 ± 0.009	0.529 ± 0.007	0.383 ± 0.003	0.726 ± 0.013
HR	1.30 ± 0.019	1.28 ± 0.004	1.24 ± 0.007	1.17 ± 0.003
CI (%)	23.2 ± 1.142	22.0 ± 0.231	19.2 ± 0.437	14.5 ± 0.23
AR (^o^)	^*∗*^	29.32 ± 0.83	23.84 ± 0.74	25.56 ± 0.89
Flow rate (g/s)	^*∗*^	4.206 ± 0.08	12.86 ± 0.02	10.50 ± 0.06
Moisture (%)	12.31 ± 0.01	10.52 ± 0.73	8.38 ± 0.73	11.21 ± 0.21

BD and TD are bulk and tapped density, respectively, HR: Hausner's ratio, CI: Carr's index, and AR: angle of repose. ^*∗*^The powders did not flow. Values are represented as standard deviation.

## Data Availability

All the data used to support the findings of this study are included within the article.
